# Measuring change in anhedonia using the “Happy Faces” task pre- to post-repetitive transcranial magnetic stimulation (rTMS) treatment to left dorsolateral prefrontal cortex in Major Depressive Disorder (MDD): relation to empathic happiness

**DOI:** 10.1038/s41398-019-0549-8

**Published:** 2019-09-03

**Authors:** Sharee N. Light, Linas A. Bieliauskas, Stephan F. Taylor

**Affiliations:** 1 Positive Affective Neuroscience Laboratory, Department of Psychology, Georgia State University, 140 Decatur Street, Atlanta, GA 30303 USA; 2 Neuropsychology Section, Department of Psychiatry, University of Michigan Medical School, 2101 Commonwealth Blvd, Suite C, Ann Arbor, MI 48105 USA; 3 Department of Psychiatry, University of Michigan Medical School, Rachel Upjohn Building, 4250 Plymouth Rd., Ann Arbor, MI 48109-2700 USA

**Keywords:** Depression, Human behaviour

## Abstract

We investigated whether repetitive transcranial magnetic stimulation (rTMS) to the left dorsolateral prefrontal cortex (DLPFC) would reduce anhedonia in a sample of 19 depressed adults (*M*_age_ = 45.21, SD = 11.21, 63% women) randomized to either active or sham rTMS. To track anhedonia, patients completed the Snaith-Hamilton Pleasure Scale (SHAPS)^1^ and a novel behavioral task called “Happy Faces,” which required patients to interpret neutral versus various intensities of positively valenced human facial expressions. Patients had to indicate dichotomously whether any degree of positive emotion was expressed. We expected that more anhedonic patients would struggle most with low intensity happy faces; often incorrectly calling them neutral. Patients also completed a self-report measure of “empathic happiness”—i.e., vicarious joy. Measures were completed pre- to post-treatment. Results indicate rTMS to DLPFC related to improvement in interpretation of subtle forms of happiness in active rTMS patients relative to sham. Furthermore, empathic happiness and anhedonia score were significantly antagonistic across all patients.

## Introduction

Anhedonia, the reduced ability to experience pleasure, is one of two possible primary diagnostic criteria that must be present for the diagnosis of Major Depressive Disorder (MDD) to be made. It occurs in up to 70% of cases of MDD^2^; however, many treatments do not directly address this symptom, and it remains a residual symptom in a fairly substantial subset (~14%) of patients who undergo treatment for an acute episode of depression^3^; but see^4^. To date, our inability to accurately identify patient’s pre-treatment level of anhedonia in an ecologically valid manner rather than based on self-report alone may be one reason that we have not been able to develop more effective treatments for MDD.

Some progress has been made toward developing a reliable and ecologically valid means to measure individual differences in anhedonia in humans. For example, Harmer et al.^5^ presented facial expressions associated with five emotions (anger, disgust, fear, happiness, and sadness) to healthy controls with and without a noradrenergic antidepressant on board. However, the faces that participants viewed were those of actors that had been averaged via use of computer graphic techniques to be between 100% emotion intensity and neutral, in 10% increments. Nevertheless, the researchers found that with administration of a noradrenergic antidepressant, these healthy adults significantly improved in their ability to process happiness. However, Langenecker et al.^6^ and Briceño et al.^7^ found that unmedicated adults with MDD showed reduced accuracy, and reaction time deficits, when perceiving/interpreting positive emotion in human faces. Importantly, the observed performance deficit related to reduced left frontal activity in these MDD patients^7^.

Although these prior studies investigated individuals with MDD and healthy controls, with an aim toward investigating their ability to interpret facial emotion, these studies had some limitations. For example, the studies used actors making posed facial configurations, e.g.,^5–8^ that were then “morphed” via computer manipulation to get variation in facial affect intensity, rather than utilizing more naturalistic face stimuli. Also, the tasks described above utilized the corpus of Ekman faces, which do not include the faces of various ethnic minority groups, lowering the generalizability of the findings.

The present study employed a new task, called the “Happy Faces Task,” in which participants had to look at human faces—an inherently rewarding stimulus—evincing neutral or varying degrees of positive emotion. The task features spontaneously generated facial affect by everyday people (including the faces of ethnic minorities) and strictly focuses on positive emotion (with neutral images interspersed), with many more positive affect trials (rather than trials of happy faces interspersed with trials of sad, angry, or fearful faces, etc.), making it a more potent elicitor of hedonic-related perceptual processing. Rather than differentiating amongst different facial expressions of discrete emotions, the task requires subjects to discern subtle differences in positive emotion only, as they are instructed to make a decision as to whether positive emotion is or is not present. The faces were divided into ‘high’ and ‘low’ intensity subtypes to further differentiate performance, and we set out to determine if a repetitive transcranial magnetic stimulation (rTMS) intervention to left DLPFC would change the perception/interpretation of this inherently positively-valenced stimulus.

Meta-analyses have demonstrated that rTMS is an effective antidepressant therapy with moderate to large effect sizes^9^. Prior research has strongly implicated the DLPFC in the pathophysiology of depression, particularly the left DLPFC^10,11^. The DLPFC and ventrolateral prefrontal regions have both been implicated in eudemonia/well-being^12^ and anhedonia^13^; particularly when attempting to up-regulate or down-regulate positive affect, respectively. Hypo-activation of DLPFC relates to the reduced experience of positive emotions^10,12^, whereas increased VLPFC activity relates to the enhanced ability to down-regulate positive emotion^13^. Prior research also indicates that those individuals with MDD who respond to antidepressant treatment show increases in activity in DLPFC pre- to post-treatment^11,14^, and decreases in VLPFC activity relate to reductions in anhedonia^13^. Therefore, it is likely that the lateral prefrontal cortex in particular is a promising region to study in relation to anhedonia; given that the left DLPFC has been found to relate to both anhedonia and well-being/positive emotionality in adults^15^ and children^16^.

The efficacy of rTMS to the left DLPFC in MDD may also lie in its indirect effects on other brain regions. Specifically, the efficacy of rTMS to the left DLPFC has been found to relate to increased negative connectivity with the subgenual cingulate^17^ and frontopolar prefrontal cortex^18^ in depressed patients. Furthermore, there is at least one rTMS study involving MDD patients that has specifically investigated the role of rTMS on aspects of anhedonia^19^. In that study, connectivity in another prefrontal region, i.e., dorsomedial prefrontal cortex—along with a region in left DLPFC—distinguished between responders and non-responders (though the dorsolateral region did not survive more stringent thresholding). This finding led the authors to hypothesize that there are at least two subtypes of depression, one of which is related to the functioning of a dorsal system in the brain that includes the dorsal sub-region of the prefrontal cortex. They called this underlying etiology a “hypoactive type” of depression, and proposed that it is responsive to rTMS to the DLPFC. Furthermore, they speculated that this dorsal “hypoactive” form of depression is characterized by preserved hedonic responsiveness^19^. The present study extends upon this previous study because the Downar et al.^19^ study had some limitations that precluded the full investigation of whether hedonic responsiveness is indeed preserved in this proposed dorsal subtype. For example, a sham control group was not included.

Individualizing rTMS treatment for particular patients may become a reality as a result of systematically investigating the effectiveness of rTMS on anhedonia, while controlling for global depression symptomatology. Ultimately, this line of research may help identify treatment protocols that are better or worse suited to target the specific symptom pattern exhibited by subgroups within the MDD population. Indeed, the efficacy of rTMS varies depending on site of stimulation, and frequency of stimulation, and not all patients who receive the treatment respond. Therefore, identifying, at the symptom level, which aspects of MDD may respond to particular rTMS protocols could be valuable.

Toward this end, we also needed to develop a behavioral task that could be used pre- to post-rTMS that is symptom-based and could be used in conjunction with self-report. For the current study, we chose to focus on the symptom of anhedonia, given its difficult to treat status, and its prominent role in the diagnosis of depression. We specifically wanted to go beyond self-report of anhedonia or use of a single item on a depression inventory. Therefore, we developed a task whereby we could track hedonic tone pre- to post-rTMS in a relatively naturalistic manner. The human face provides important information for decoding the mental states of others, and may be one very important inlet for the experience of empathic happiness (i.e., the ability to vicariously experience positive affect in response to the joy of another), which provides a route by which positive emotion can get under the skin (so to speak) and be transmitted from one person to another.

Thus, we hypothesized that a keen ability to detect, interpret, and subjectively experience positive emotion from viewing the positive affect displays from the facial region of others, would predict a person’s proneness to experience day-to-day subjective positive emotion themselves and would also relate to their ability to experience empathic happiness; and this process would be linked to the functioning of the lateral PFC, given its role in trait positive emotionality, e.g.,^20,21^. We predicted that relative to baseline (pre-treatment), treatment with rTMS targeting the left DLPFC would lower the patient’s threshold for detecting, interpreting, and appreciating positive affect on the face of others. In particular, it was expected that the “low” intensity happy face trials from the Happy Faces Task would be a much more sensitive measure of anhedonia in this MDD population; with patients more often labeling “low” intensity happy faces (i.e. faces with subtle smiles) as neutral at baseline, and this response normalizing with treatment. We did not expect the “high” intensity happy faces (i.e. frank smiles) score to change with treatment.

We also hypothesized that greater levels of self-reported “empathic happiness” would relate to lesser anhedonia. Studies support an association between empathy and positive emotion, e.g.,^16^. For example, compared with a control group, people who completed compassion training—a construct related to empathy—reported experiencing increased positive emotion^22,23^. This increase was associated with increased activity in DLPFC and nucleus accumbens^23^. To formally address this hypothesis, in addition to our novel behavioral task of anhedonia (i.e. the Happy Faces Task), two self-report measures specifically geared toward tracking anhedonia and vicarious positive emotion were administered to patients: the Snaith-Hamilton Pleasure Scale (SHAPS)^1^ and the Empathic Happiness subscale of the Light-Moran Positive Empathy Scale^24^, respectively. These measures are both self-report measures and provided an additional means to measure subjective change in positive affectivity in our patients with treatment.

The Snaith-Hamilton Pleasure Scale specifically measures anhedonia, and the Empathic Happiness subscale of the Light-Moran Positive Empathy Scale measures the ability to vicariously experience positive emotion. Thus, our secondary hypothesis was that change in empathic happiness level with treatment would relate to change in anhedonia level; primarily because any change in the ability to enjoy obtained rewards first-hand may relate to the ability to do so vicariously. Together, the self-report measures and the behavioral task enabled us to determine whether the actual increased ability to detect, interpret, and appreciate positive emotion by looking at the facial region of others relates to rTMS, taking subjective change in anhedonia into account.

## Method

### Study overview

This was a randomized, double-blind study of the effects of rTMS. All patients underwent a psychiatric assessment (a MINI-SCID and psychiatric interview) before study enrollment. All participants eventually received rTMS treatment, but at study entry, there was a 50–50 chance that each patient would be randomized to a sham treatment for the first 4 weeks. Sample size was based on previous rTMS studies investigating similar endpoint measures, e.g., Downar et al.^19^.

#### Inclusion criteria

included the following: male or female, age 22–65 diagnosed with MDD with failure of at least one antidepressant treatment at adequate dose/duration or failed to achieve adequate dose/duration due to intolerable side effects, and on a stable dose of medication for at least 4 weeks prior to TMS therapy. The Montgomery-*Å*sberg Depression Rating Scale – Clinician version (MADRS)^25^ was used to define “caseness,” and a score at baseline on the MADRS of 18 or greater was required for study entry.

#### Exclusion criteria

included the following: diagnosed with a psychotic, bipolar, obsessive-compulsive, or post-traumatic stress disorder, active substance abuse or met criteria for substance use disorder in the past 6 months, have or have had in the past a medical or a neurological condition that could affect brain function or risk of seizure, including stroke, epilepsy, or a closed head injury, presence of an implanted device or metal in the body which would prevent fMRI scanning, pregnant or trying to get pregnant, failed to respond to an adequate course of electroconvulsive therapy (ECT), previous treatment with rTMS, serious suicidal ideation/behavior, and current depressive episode longer than 5 years in duration.

The study was approved by the Institutional Review Board of the University of Michigan. A full report of the clinical study is published elsewhere^26^; here, we focus on the subset of subjects who completed a paper-and-pencil anhedonia measure and the (behavioral) Happy Faces Task.

### rTMS

The protocol was a sham-controlled, randomized, double-blinded study. Subjects who received sham stimulation had the option to receive active rTMS in the second, open-label phase of the study. After initial screening and assessment, subjects entered phase 1, consisting of motor threshold obtained using visual identification of thumb twitch and initiation of treatment. In this blinded phase, subjects had 20 sessions of TMS therapy or sham treatment, delivered 5 days per week. TMS treatments were delivered at 10 Hz frequency at 120% of motor threshold and 3000 pulses/session to the dorsolateral prefrontal cortex, at a location determined by neuronavigation from the MRI session. TMS therapy was delivered with the NeuroStar XPLOR system in research mode (detailed in Taylor et al.^26^). One coil was active, while the sham coil was identical in shape and weight as the active coil, but did not deliver any magnetic energy. A speaker on the coil gantry delivered a 10-Hz pulsed sound mimicking acoustic characteristics of the active coil. At the end of phase 1, either 5 taper TMS sessions over 2 weeks for those in the active arm, or 20 sessions plus 5 taper sessions, were also delivered over 2 weeks.

The primary endpoint was the Happy Faces Task score (Accuracy and Reaction Time) and the Empathic Happiness subscale score from the Light-Moran Positive Empathy Scale^24^. We also administered the SHAPS^1^ as an additional self-report measure of anhedonia (and to help make qualified statements about the effect of rTMS on subjectively experienced versus objectively measured anhedonia), and the MADRS to account for overall depression severity in our analyses. Assessment utilizing the MADRS and SHAPS occurred weekly for the 20 sessions of TMS treatment; and again at the end of the last taper session for the MADRS; but only at baseline and at session 20 for the SHAPS, Empathic Happiness subscale, and the Happy Faces Task. Planned analysis of the primary endpoint scores was a repeated measures model.

### Behavioral measures

#### Montgomery-Åsberg Depression Rating Scale – Clinician version

This 10-item, clinician-rated scale includes questions on sadness, inner tension, reduced sleep, reduced appetite, concentration difficulties, lassitude, inability to feel, pessimistic thoughts, and suicidal thoughts. The score range is 0–60 points, with higher scores indicating more severe depression^25^.

#### Snaith-Hamilton Pleasure Scale

This 14-item self-report scale assesses the respondent’s ability to feel pleasure in response to stimuli that typically elicit positive emotion. Items are measured on a Likert-scale from 1 (definitely agree) to 4 (strongly disagree)^27^. The SHAPS covers four domains: interests/pastimes, social interaction, sensory experience, and food/drink. The inter-item reliability (Cronbach’s alpha) for this test is 0.857^1,28^. Higher scores indicate greater anhedonia.

#### Empathic Happiness subscale of the Light-Moran Positive Empathy Scale

This 8-item self-report subscale measures empathic happiness (subscale Cronbach’s alpha = 0.87). Items are measured on a Likert-scale from 1 (not at all true) to 7 (extremely true). The overall inter-item reliability (total Cronbach’s alpha) for this measure is 0.92^24^. Higher scores indicate greater empathic happiness.

#### The Happy Faces Task

**Fig. 1:**
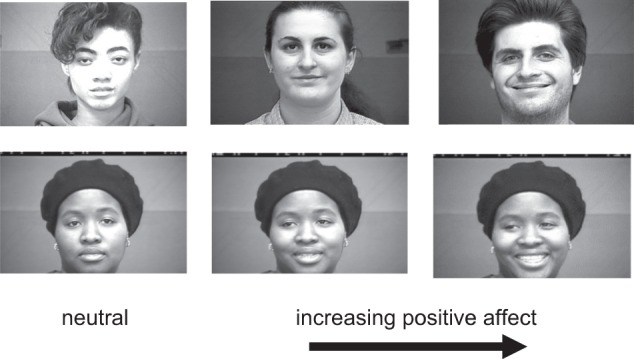
Examples of Happy Faces Task stimuli. During the “Happy Faces” Task, participants viewed randomly ordered pictures of human faces displaying either neutral affect or varying degrees of positive affect

This task was designed as an initial and viable means by which to ascertain the level of anhedonia present pre- to post-treatment. During the task, patients are asked to look at human faces evincing varying degrees of positive emotion, based on Ekman’s fine-grained FACS coding, with a balance of gender (see Fig. 1 for an example of stimuli). There were 20 low intensity trials, 33 high intensity trials, and 22 neutral trials interspersed. Determination of low versus high intensity group inclusion was made based on FACS units present on the face. Specifically, for low intensity items, only zygomaticus activity could be present, whereas for high intensity items orbicularis and zygomaticus activity was necessary for inclusion. Faces were taken from the well validated Cohn-Kanade dataset^29,30^, which includes hundreds of images of human faces expressing spontaneous neutral and varying degrees of spontaneous positive emotion^31,32^. Participants were instructed to indicate by button press whether any positive emotion was present on the face they were viewing in “yes” or “no” format. Importantly, the face remained on the screen until the participant made a response, and their reaction time was recorded. Task duration was approximately 10 min for each participant. Participants’ performance on the task was tracked pre- to post-treatment with rTMS treatment to the left DLPFC (20 sessions of treatment) to determine whether their ability to detect, interpret, and appreciate positive emotion via the face would change as a function of treatment.

### Analysis plan

Planned analyses included a repeated measures model looking for the effect of rTMS specifically on MADRS score, and the effect of rTMS on Happy Faces performance (i.e., Accuracy, Reaction Time), co-varying for baseline *MADRS* score (to control for depression severity), age, self-reported anhedonia change (i.e., SHAPS Time 1 minus Time 2 score), and gender. Specifically, Low Intensity Happy Face Trial Accuracy and Reaction Time at Time 1 versus Time 2 were included as a repeated measures outcome variable. Group (i.e., Sham versus Active rTMS patients) was the between subjects variable. MADRS score at screening was used as a co-variate in the model to control for overall depression severity. Change in subjective (i.e., self-reported) anhedonia, i.e., measured via the Snaith-Hamilton Pleasure scale score at Time 2 minus Time 1, was also used as a co-variate in the model to enable us to determine whether change in Happy Faces Accuracy (or Reaction Time) would be significant above and beyond subjectively-reported anhedonia. Finally, age and gender were also included in the model as covariates. Overall, the model evaluated whether the mean of Happy Faces Accuracy and Reaction time scores were equal across Time 1 versus Time 2, while statistically controlling for the effects of age, gender, baseline depression severity, and change in subjective anhedonia.

Furthermore, regression analyses were conducted to look at (a) *SHAPS* score (or change in SHAPS score from Baseline/Time 1 to 20 Sessions Later/Time 2), predicting Low Intensity Happy Face trials score (Accuracy and/or Reaction Time), (b) *Empathic Happiness* subscale score predicting Low Intensity Happy Face trials score (Accuracy and/or Reaction Time), and (c) *Empathic Happiness* subscale score predicting *SHAPS* anhedonia score at Baseline and 20 Sessions Later.

## Results

### Sample descriptive statistics

Nineteen participants completed both pre- and post- aspects of the “Happy Faces” Task and had complete pre- and post- self-report data (Table 1): attrition and incomplete or incorrect completion of self-report forms precluded data analysis of all study participants as reported in Taylor et al.^26^. Therefore, 19 subjects were entered into the repeated measures ANOVA (see section 1.2). In all, 97% of the sample identified themselves as non-Hispanic white, with only one participant identifying as Asian.

**Table 1:** Baseline demographic and clinical variables by depression sub-group

Variable	Active rTMS group (*N* = 8)		Sham group (*N* = 11)		Significance (*p*-value)
	Mean (SD)	Min-Max	Mean (SD)	Min-Max	
Age (in years)	44.13 (13.32)	24–60	50 (8.75)	40–61	0.404
Gender	7 women	1 man	5 women	6 men	–
MADRS Score (Screen)	23.75 (5.06) moderate severity	18–30	21.72 (3.87) moderate severity	18–31	0.337
Empathic Happiness Subscale Score at Time 1	35.12 (8.82)	22–50	31.91 (7.58)	19–45	0.406
Snaith-Hamilton Pleasure Scale Score at Time 1	32.13 (4.58)	26–40	36 (4.49)	28–43	0.083
Low Intensity Happy Faces Percent Correct Score at Time 1	61.25% (20%)	30–80%	63.18% (27%)	5–90%	0.866

There were no significant differences between active and sham groups in terms of depression severity (measured via the MADRS), race/ethnicity, age, empathic happiness, or anhedonia score—measured behaviorally or via self-report—at baseline/Time 1 (all *p*’s > 0.05; Table 1).

### Validity of the “Happy Faces Task”

The Happy Faces Task demonstrated good scale reliabilities with a Cronbach’s alpha value = .83 for the pre-treatment test, and 0.75 for the post-treatment test. Test-Retest Reliability was relatively low—as expected for a scale that is measuring actual change across time—and equaled 0.66. For the pre-test, item-total correlations ranged from −0.70 to 0.65. For the post-test, item-total correlations ranged from −0.38 to 0.64. An ANOVA revealed no differences in Accuracy as a function of the race of the stimulus (*p* = 0.564).

### Anhedonia reduction (as reflected by Happy Face Performance) in rTMS active versus sham participants

Based on the 19 participants with complete Baseline (Time 1) and 20 Sessions Later (Time 2) data (Table 1), and in line with prediction, there was a significant between-subject effect for SHAPS score across Time 1 to Time 2 (*p* = 0.05), and a significant within-subject Time × Group effect (*p* < 0.05).

**Fig. 2:**
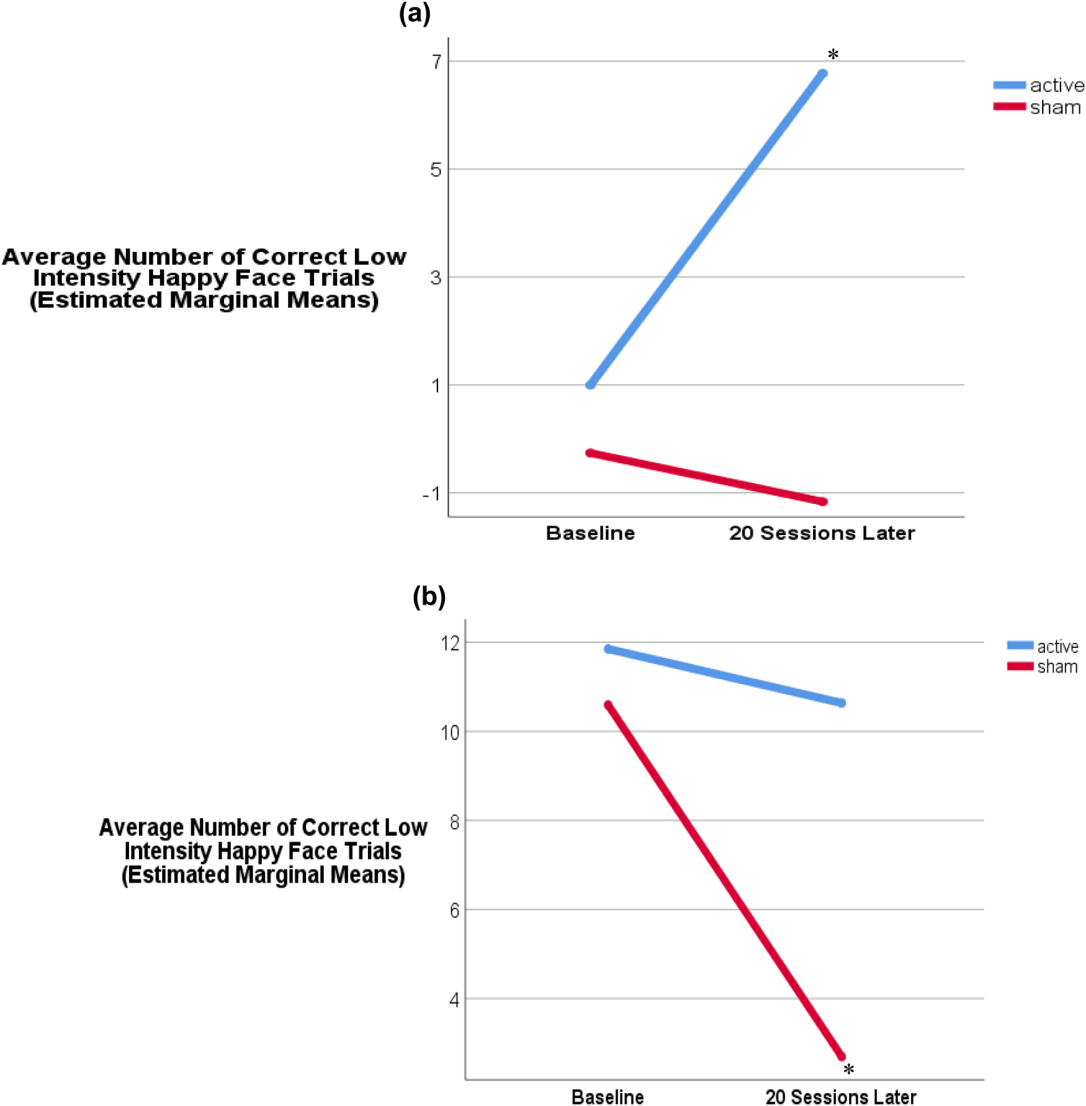
Significant Time × Low Intensity Happy Faces Accuracy interaction. **a** Covariates appearing in the model are evaluated at the following values: MADRS score at screening = *Mean*, SHAPS Decline = 0, age = mean years. **b** Covariates appearing in the model are evaluated at the following values: MADRS score at screening = mean, SHAPS Decline = 10, age = mean years

Inspection of univariate analyses provided a more nuanced picture of these results. Specifically, degree of change in subjective anhedonia (as measured by the SHAPS score at Time 1 minus Time 2) influenced how Low Intensity Happy Faces Accuracy score changed across time. This was present when controlling for MADRS change, so this effect cannot be attributed to the overall change in depression severity; *p* = 0.05). In other words, the relationship between Time and Low Intensity Happy Faces Accuracy score varied depending on the degree of SHAPS decline (but not MADRS decline). Importantly, this means that the significant Time × Group interaction reported above varied specifically as a function of SHAPS score (see Fig. 2a, b). For example, active rTMS participants that showed a meaningful decline in SHAPS score across Time 1 to Time 2 were also those individuals who tended to demonstrate an increase in the number of correctly identified Low Intensity Happy Faces across time; whereas sham participants actually got worse at the task across time (compare Fig. 2a, b).

**Fig. 3:**
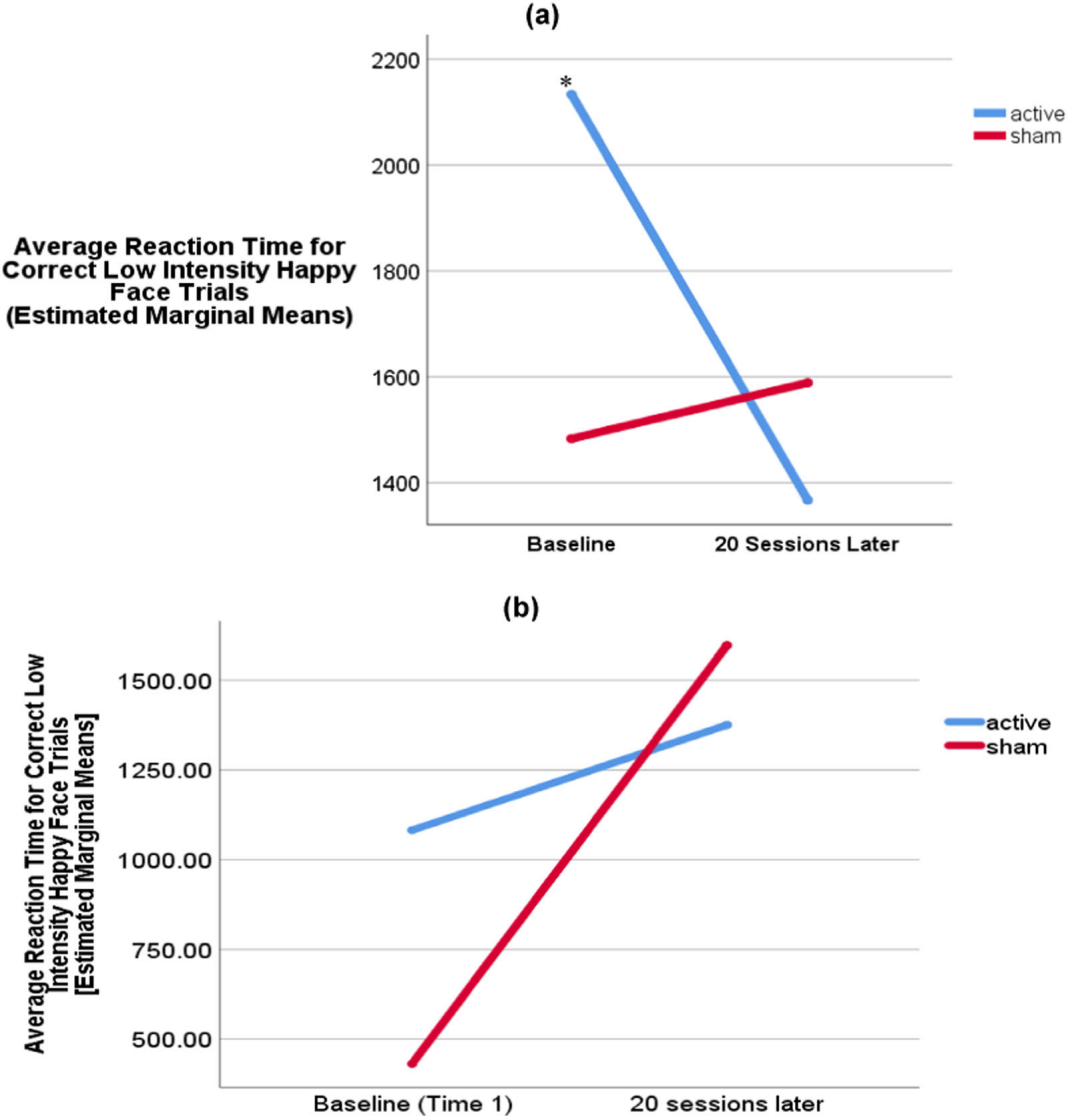
Significant Time × Low Intensity Happy Faces Reaction Time interaction. **a** Covariates appearing in the model are evaluated at the following values: MADRS score at Baseline = 27, SHAPS Decline = 10, age = mean years. **b** Covariates appearing in the model are evaluated at the following values: MADRS score at Baseline = 18 (minimum for study entry), SHAPS Decline = 10, age = mean years

In addition, a trend level Time × Low Intensity Happy Faces — Reaction Time effect was observed (*p* = 0.08; Fig. 3a, b). As such, the relationship between Time and Low Intensity Happy Faces Reaction Time score varied at a trend level depending on the severity of depression (as measured by the MADRS) at Baseline (i.e., Time 1). This means that the significant Time × Group interaction varied specifically as a function of MADRS score (see Fig. 3a vs. b). Active rTMS participants that were more severely depressed at Baseline were also those individuals who tended to get faster at identifying Low Intensity Happy Faces across time; whereas severely depressed sham participants did not show a significant change (Fig. 3a).

There was a significant Time × Gender effect for Correct Low Intensity Happy Faces trials (but not Reaction Time), such that women became more accurate across time relative to men. Finally, and very importantly, there was a significant Time × Group effect (*p* *<* 0.01) for MADRS score, such that individuals in the active treatment group demonstrated an overall decline in MADRS score across time, whereas sham participants did not; and this was independent of anhedonia decline (i.e., SHAPS change score = 0).

### Total sample results

When not separating the sample into an active rTMS and sham group, several more key findings emerged. First, regarding correlations between key Time 1/baseline variables (accounting for depression severity—i.e., MADRS screening score included in the model), empathic happiness at Time 1 negatively correlated with SHAPS score at Time 1 (*r* = −0.796, *p* < 0.01), indicating that individuals higher on empathic happiness had a lower level of anhedonia. Also, empathic happiness at Time 1 positively correlated with number of correct Low Intensity Happy Face trials at Time 1 (*r* = 0.662, *p* < 0.05).

In addition, regarding correlations between key “change” scores (controlling for severity via the MADRS), the greater the increase in Empathic Happiness score from Time 1 to Time 2, the greater the decrease in SHAPS score (i.e., reduced anhedonia) from Time 1 to Time 2 (*r* = 0.549, *p* < 0.05).

#### Performance on the Happy Faces Task and change in anhedonia

The greater the reduction in SHAPS anhedonia score from Baseline (Time 1) to 20 Sessions Later (Time 2), the greater the number of correctly identified *Low Intensity* Happy Faces at (a) Time 1 (*p* < 0.05; Adjusted *R*^2^ = 33%) and (b) Time 2 (*p* < 0.05; Adjusted *R*^2^ = 20%), even with MADRS score entered as a co-variate in each model. Furthermore, the greater the reduction in SHAPS anhedonia score from Time 1 to Time 2, the greater the number of correctly identified *High Intensity* Happy Faces at Time 1 (*p* < 0.05; Adjusted *R*^2^ = 24%), but not at Time 2 (*p* = 0.123); even with MADRS score entered as a co-variate in each model. Importantly, the difference in magnitude between the Low Intensity Happy Face model and the High Intensity Happy Face model is significant (i.e. Adjusted *R*^2^ = 33% versus 24%, *p* < 0.05).

#### Trait Empathic Happiness Score and Happy Face Performance

A greater increase in patient’s trait Light-Moran Empathic Happiness subscale score from Time 1 to Time 2 predicted a greater number of correctly identified Low Intensity Happy Faces at Time 2 (*p* < 0.05; Adjusted *R*^2^ = 15%), even with MADRS score entered in the regression as a co-variate. This effect was absent when looking at High Intensity Happy Face trials (*p* = 0.367).

#### Direct relation between Trait Empathic Happiness and anhedonia

**Fig. 4:**
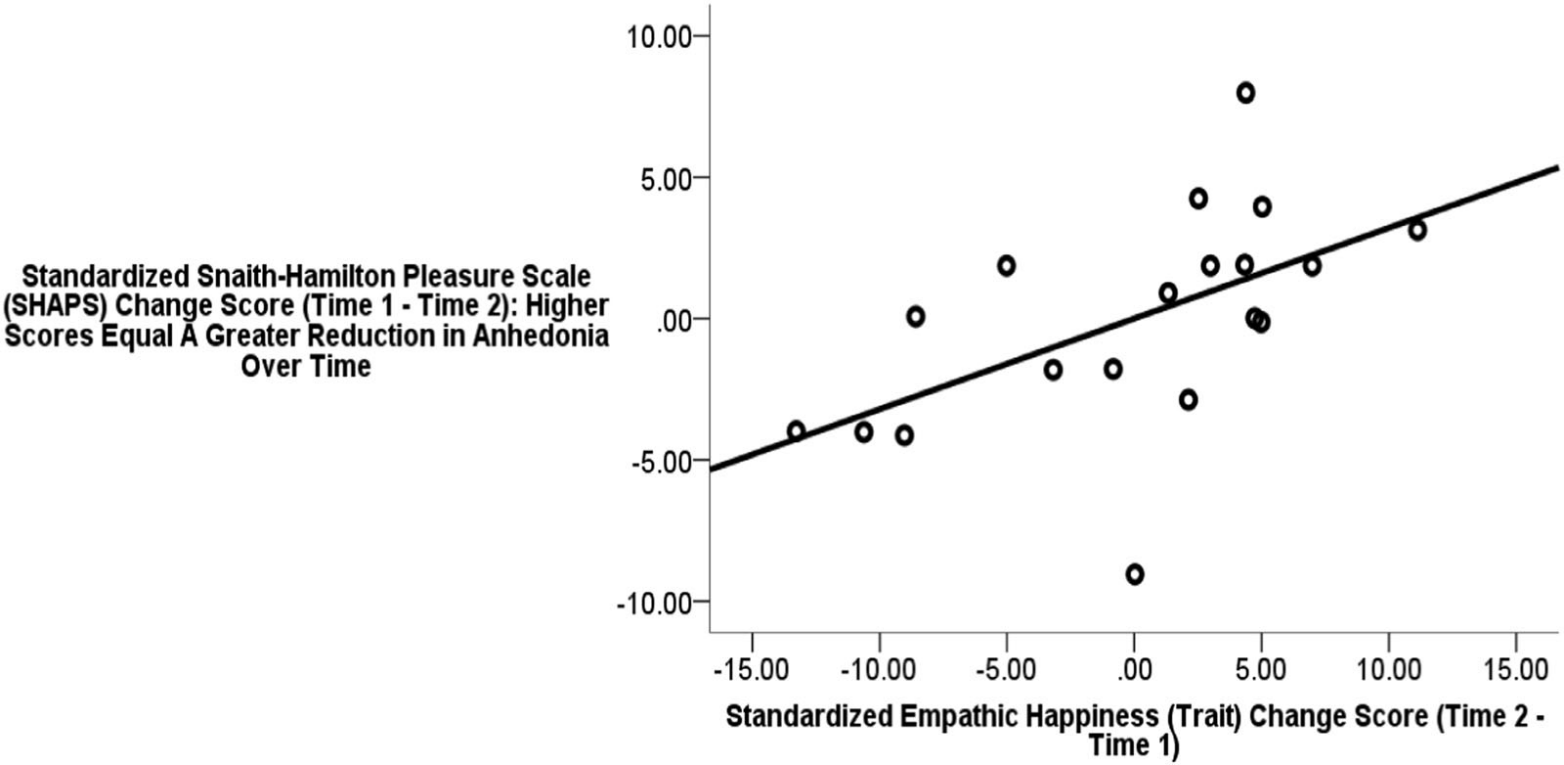
Partial regression plot. The greater the increase in trait Light-Moran Empathic Happiness subscale score from Time 1 to Time 2, the greater the increase in hedonic tone (i.e. the greater the reduction in anhedonia) from Time 1 to Time 2, even with MADRS screening score entered as a co-variate (*ß* = 0.563, *t* *=* 2.63; *p* < 0.05; Adjusted *R*^2^ = 22%)

The greater the increase in trait Light-Moran Empathic Happiness subscale score from Time 1 to Time 2, the greater the increase in hedonic tone (i.e. the greater the reduction in anhedonia) from Time 1 to Time 2—even with MADRS score entered in the regression as a co-variate (*p* < 0.05; Adjusted *R*^2^ = 22%; Fig. 4).

#### Post-Hoc mediation analysis

We also investigated the extent to which change in empathic happiness score mediates the relationship between self-reported anhedonia change (SHAPS score) and performance on the Happy Faces Low Intensity Trials (Accuracy) at Time 2. The mediation analyses indicated full mediation based on the Baron & Kenny^33^ method. However, given our small sample size, it is most likely that there is *partial* mediation based on the reduction in the beta-coefficient from 0.549 to 0.40 (*p* *<* 0.01) when the mediator is included in the model; even though the *statistical significance* of the model indicates full mediation.

## Discussion

The results suggest that MDD patients have greater difficulties with detecting, interpreting, and appreciating low intensity positive affective displays versus high intensity positive affective displays; and a greater increase in trait empathic happiness relates to reduction in anhedonia (measured both via self-report and behaviorally via the Happy Faces task). This indicates that empathic happiness as a construct may be a valuable adjunctive tool for non-invasively predicting treatment response in at least a subset of MDD patients. In addition, our data suggest that empathic happiness itself is a mutable construct, as empathic happiness score changed over time, and the magnitude of change predicted change in anhedonia across time, and predicted ultimate outcome of Low Intensity Happy Face performance at Time 2. Ultimately, our mediation analysis points to change in Empathic Happiness driving the relationship between change in anhedonia and accuracy on the Low Intensity Happy Face task at Time 2.

Furthermore, our data suggest that treatment with rTMS to the left DLPFC may play a role in reducing behaviorally measured anhedonia across time in MDD patients; i.e. there was a significant interaction between group (active versus sham) and our behavioral anhedonia measure, such that patients receiving the active treatment showed improved performance on Low Intensity Happy Faces trials (and also showed an overall reduction in MADRS score) if they also reported greater change in subjective anhedonia; and this implicates actual behavioral change in responsivity to life-like subtle positive socio-cognitive stimuli (i.e., mildly happy faces) that we encounter on a daily basis, with the active treatment. A prior fMRI study utilizing the Happy Faces task found that DLPFC activity and nucleus accumbens shell activity specifically related to better performance on this task^21^. When combined with the current findings, this may suggest a fronto-striatal basis for rTMS impact on anhedonia symptomatology.

Regarding the relatively specific effect of rTMS on low intensity happy face performance versus high intensity happy face performance, consistent with our hypothesis, we believe our data provide straightforward evidence that individuals with depression do indeed experience consummatory anhedonia, though this can be subtle, as reflected by relative poorer performance when identifying low intensity positive faces relative to high intensity positive faces. In other words, our results provide preliminary evidence to suggest that—at least in terms of complex socio-cognitive stimuli—it takes presentation of more intense positive stimuli for individuals who are depressed to appreciate the hedonic quality of that stimulus, and this correlates with not only their own subjective happiness, but their ability to feel vicarious happiness.

Overall, our Happy Faces Task is a synergistic objective behavioral marker/index of a very specific socio-cognitive aspect of anhedonia; and its use may enhance our understanding of the nuances of anhedonia above and beyond simply measuring change in patient’s self-reported/subjectively experienced difficulties with anhedonia-- and this is an important advancement in the field. These data highlight the idea that MDD patients may only be perceiving a truncated portion of neuro-typical happiness-generating stimuli in the world, similar to how neglect patients only perceive a select portion of the visual-spatial world, thus rendering it impossible for them to interpret and subjectively experience joy. These preliminary findings offer some indication that rTMS to the DLPFC is impactful on this important symptom.

Limitations of the current study include small sample size, lack of an ethnically diverse patient sample, uneven gender across groups, lack of a non-depressed control group, and somewhat limited range of depression severity (i.e. mild to moderate severity depression only). Although we have linked hedonic processing with the left DLPFC, because this is where the rTMS coil stimulates, we cannot say whether or not the effect on happiness perception was mediated by the DLPFC. In fact this region’s connectivity to frontopolar PFC^34^ and subgenual cingulate^18^, and the known impact of rTMS to DLPFC to induce dopamine release in the caudate^35^, could be relevant pathways to reduction of anhedonia symptomatology. It is even possible that another brain area, also affected by rTMS, could be mediating this effect on face processing. Also, participants saw the same faces pre- to post-treatment, though face presentation was randomized each time. Therefore, there may have been potential for practice effects (but the inclusion of a sham group helps to extirpate this concern).

In conclusion, rTMS to the left DLPFC in a depressed patient sample related to significant improvement in the recognition/interpretation of real-life subtle positive emotional cues in MDD patients, when the patient also self-reported a concurrent decline in anhedonia. Furthermore, across all patients (sham patients and active rTMS patients), greater increases in empathic happiness appears to be positive prognostically for anhedonia reduction across time. What is more, empathic happiness appears to be mutable, showing significant increases across a 4-week span.
